# Use of systemic hormonal contraception and risk of attempted suicide: a nested case–control study

**DOI:** 10.1007/s10654-024-01155-z

**Published:** 2024-09-03

**Authors:** Elena Toffol, Timo Partonen, Oskari Heikinheimo, Anna But, Antti Latvala, Jari Haukka

**Affiliations:** 1 Department of Public Health, Clinicum, University of Helsinki, Helsinki, Finland; 2 Department of Public Health and Welfare, Finnish Institute for Health and Welfare, Helsinki, Finland; 3 Department of Obstetrics and Gynaecology, University of Helsinki and Helsinki University Hospital, Helsinki, Finland; 4 Institute of Criminology and Legal Policy, University of Helsinki, Helsinki, Finland

**Keywords:** Attempted suicide, Hormonal contraception, Women, Register

## Abstract

Evidence regarding the role of hormonal contraception (HC) as a risk factor for attempted suicide is inconclusive. Thus, this study aimed to assess the associations of use of different types of systemic HC with the risk of attempted suicide in women aged 15–49 years. Data on a population-based cohort (n = 587,823) of HC users and non-users in 2017 was derived from national registers in Finland. In a nested case–control design we examined the risk of attempted suicide in relation to current HC use (past six months) via multivariable conditional logistic regression models. During the follow-up (from 2018 to 2019) there were 1.174,346 million person-years of which 818 cases of suicide attempts were observed (incidence rate: 0.70 per 1000 person-years). Use of HC, especially combined hormonal contraceptives, was not associated with a higher risk of attempted suicide compared to non-use (OR 0.68, 95% CI 0.45–1.02) after controlling for marital status, socioeconomic status, education, chronic diseases, recent delivery, recent psychiatric hospitalizations, and current use of psychotropic medications. In women without psychiatric history, current HC use (OR 0.73, 95% CI 0.58–0.91), especially ethinylestradiol-containing preparations (OR 0.54, 95% CI 0.40–0.73), was associated with a lower risk of attempted suicide. After adjusting for recent psychiatric hospitalizations and use of psychotropic medications, current use of progestin-only preparations was not associated with attempted suicide. In conclusion, current HC use was not associated with an increased risk of attempted suicide in fertile-aged women.

**Supplementary Information:**

The online version contains supplementary material available at 10.1007/s10654-024-01155-z.

## Introduction

The global age-standardized suicide rate in 2019 was 9.0 per 100,000 persons (12.6 in males and 5.4 in females) [1], which corresponds to more than 700,000 suicidal deaths every year. It is estimated that for every adult who dies by suicide, there are more than 20 people who attempt suicide [2]. Given that a prior suicidal attempt is the major risk factor for a death from suicide [2], it is evident that suicidal behaviour represents a major public health problem.

Attempted suicides (AS) are two to three times more common in women than in men, especially among the young age-groups [3, 4]. In addition to current or previous psychiatric disorders, many other physiological, psychological or social factors contribute to the risk of AS. Hormonal contraception (HC) as a risk factor for AS has gained particular attention in recent years, especially with respect to young, fertile-aged women [5, 6]. This is of general interest, given that around 970 million of fertile-aged women use contraception globally [7], and approximately a third of them use a hormonal method, the pill being the most commonly chosen option [8]. However, results appear inconsistent [9], as some studies have shown a reduced risk or no association between the use of contraception and suicidal risk [10–14]. For example, in a 14-year longitudinal US study of women aged 25–34 years, Keyes et al. [13] found a reduced (by 62%, 95% CI 5%-86%) risk of past-year AS among HC users compared to users of non-hormonal methods. Similarly, a recent Swedish register-based study reported no or a lower risk of AS in relation to HC use in women with premenstrual disorders and with psychiatric disorders (incidence rate ratio in between-individual analyses: 0.77, 0.39–1.50) or without psychiatric disorders (incidence rate ratio 0.70, 0.40–1.24) [14].

Different types of HC are known to have different profiles of side effects and adverse events, including mood effects, mostly depending on the types and doses of oestrogens and progestogens [15]. However, to the best of our knowledge, only few recent studies have examined the associations of different types and doses of HC with the risk of suicidal behaviour [5, 6, 14], with partly inconclusive results.

Thus, the aim of this study was to assess the associations of the current use of HC with the risk of AS. Specifically, we examined the risk of AS in relation to different types of systemic hormonal contraceptives in a large cohort of fertile-aged women using recent high-quality registry data from Finland.

## Method

### Study population and design

This study was part of a larger register-based study of HC use in Finland [16]. Briefly, the sampling population was selected, based on the unique personal identification number that is given at birth or at immigration to each person permanently residing in Finland, as inclusive of all fertile-aged women (15–49 years) with at least one redeemed prescription for HC (Anatomical Therapeutic Chemical -ATC- codes: G02B, “contraceptives for topical use”; G03A, “hormonal contraceptives for systemic use”; G03HB, “antiandrogens and oestrogens”) in 2017 (n = 294,445) according to the Prescription Centre in the Kanta Services (https://www.kanta.fi/en/what-are-kanta-services). Prescriptions of HC in Finland are usually made by medical doctors at primary health care centres or by gynaecologists at family planning services and at the private sector; they are preceded by routine screening of contraindications of HC use, and based on a national guideline on contraception [17]. A 1:1 matched (by age and municipality of residence) reference group of women with no redeemed HC prescriptions in 2017 (HC non-users) was also selected. After exclusion of 89 women with a prescription for emergency contraception (ATC code “G03AD”, usually available without prescription in Finland), and of their matched control individuals, a final population of 294,356 HC users and a same-sized reference group were retained for the analyses. Through the Prescription Centre the HC use of all these women was followed-up until the end of 2019.

Given that a previous AS is per se a known risk factor for further suicidal behaviour, to obtain estimates of HC associations with suicidal risk as unconfounded as possible, the 889 prevalent AS cases (i.e., with an endpoint event in 2016 and 2017, before the start of follow-up) were excluded. The resulting cohorts of HC users and non-users (n = 587,823), which altogether included more than half of all fertile-aged women living in Finland in 2017, were used as a sampling frame for a nested case–control study, which explored the risk of AS related to current (i.e., in the six months before the event) HC use. Because register records of HC prescriptions were not available in Finland before 2017, the follow-up started on 1 January 2018 and ended on 31 December 2019, with a maximum length of follow-up of two years. A first hospitalization or visit to specialized outpatient care (as recorded in the Care Register for Health Care) due to attempted suicide (International Classification of Diseases, Tenth Revision, ICD-10 diagnosis “X[6-7]|X8[0–4]”) or a primary health-care contact due to attempted suicide (as recorded in the Register of Primary Health Care visits; ICD-10 diagnosis “X[6–7]|X8[0–4]” or International Classification of Primary Care, ICPC, “P77”) were considered as primary endpoint events. All the recorded events were based on clinical diagnoses set by medical doctors. The follow-up was discontinued at death, emigration from Finland or end of the two-year follow-up period. For each incident AS case we selected four controls, matched by year of birth.

### The register data and variables

Information on sociodemographic characteristics of all the study members on 31 December 2017 (age, municipality of residence, civil status, socioeconomic status, highest level of education) was retrieved from Statistics Finland. Information about recent deliveries (within the previous two years) was gathered from the Medical Birth Register. Data on special reimbursement rights for chronic diseases (diabetes, multiple sclerosis, epilepsy, severe psychiatric disorders, connective tissue diseases, ulcerative colitis or Crohn’s disease) were obtained from the Social Insurance Institution of Finland. Data on recent (in the past two years) hospitalizations or visits to specialized outpatient care due to psychiatric disorders (ICD-10 codes F00-F99) were gathered from the Care Register for Health Care. In addition to selection of the initial study population based on redeemed HC prescriptions in 2017, the Prescription Centre was used to gather information on their HC use in the period 2018–2019. Only ATC codes with at least five individuals in all categories were used in statistical analyses. Moreover, from the Prescription Centre we gathered information on use of psychotropic medications in the 360 days before the event (ATC codes: N05A, antipsychotics; N05B, anxiolytics; N05C, hypnotics and sedatives; N06A, antidepressants; N06B, psychostimulants, N06C psycholeptics and psychoanaleptics in combination). Use of each substance (hormonal contraceptives and psychotropic drugs) was defined as two or more redeemed prescriptions in a 180-day period.

In this nested case–control study, for each HC substance we defined a categorical variable as follows: non-user (no use in the 180 days before the AS event) and current user (use in 1–180 days before the event). Moreover, to exclude a healthy user bias, an additional categorical variable defining current (in the 180 days before the event), former (any time from 1.1.2017 up to 180 days before the event) and never (in the period 2017–2019) use was created. The HC methods of interest and available in Finland in 2018 are summarized in Table S1. Contraceptive implants and the levonorgestrel-releasing intrauterine system (but not transdermal patches and vaginal ring) were excluded, because they are often provided free-of-charge by single communities, thus not necessitating individual prescription (and as such they are not completely covered in the Prescription Centre database). Additionally, levonorgestrel-only containing oral contraceptives were excluded from further analyses because of the small numbers of users and of associated AS cases.

### Statistical analyses

To take into account matching, we utilized conditional logistic regression, with group of utilized HC (current use *vs*. non-use) as the main predictor. In addition to a univariate model, we performed Model 1, controlled for marital status, socioeconomic status and education; Model 2, further controlled for chronic diseases (indicator of chronic diseases before start of follow-up) and recent (in the previous six months or in the previous two years) delivery; and Model 3, which is Model 2 further adjusted for recent (in the previous six months or in the previous two years) psychiatric hospitalizations and use of psychotropic medications in the 360 days before the event (Figure S1). Because the case and control groups were matched by year of birth, age was not included as covariate in the models; rather, age-stratified analyses were performed. Sensitivity analyses were conducted by only considering AS cases recorded in the Care Register for Health Care (i.e., serious AS requiring hospitalization or specialized outpatient care). Additionally, to further take psychiatric disorders into account, the analyses were repeated after exclusion of women with special reimbursement rights for severe psychiatric disorders (including severe psychosis, depression with psychotic features, mania and severe bipolar disorder) before the start of follow-up. Moreover, analyses were also conducted in groups stratified by psychiatric history (those with and without a recent hospitalization due to psychiatric disorders-including eating disorders, use of psychotropic drugs or reimbursement rights for severe psychiatric disorders at baseline). Thus, psychiatric disorders as indicated by a care episode due to psychiatric problems or use of antidepressants were both considered as confounders, and additionally tested as moderators of the relationship between HC use and suicide attempt. Additionally, to exclude a healthy user bias, unadjusted and adjusted (Model 2) conditional regression analyses were conducted with the main predictor being current *vs.* former *vs.* never HC use.

Finally, to specifically assess the risk of AS in relation to HC use in a high-risk population, such as those with a previous history of AS, sensitivity analyses were conducted solely in the group of women who had attempted suicide before the starting of the follow-up (i.e., between 01 January 2016 and 31 December 2017).

All the analyses were performed with R software version 4.0.5, packages “Epi” (version 2.44) and “survival” (version 3.2.13) [18–21].

## Results

During the follow-up 1,174,346 person-years were cumulated and 818 AS cases observed, with an overall incidence rate (IR) of 0.70 (95% CI 0.63–0.83) per 1000 person-years. The incidence of AS decreased with age, with the highest IR in the age group 15–19 years (1.62, 95% CI 1.42–1.83) (Table S2).

The nested case–control study for AS built on this cohort consisted of altogether 4090 women (818 AS cases and 3272 controls). Compared to the controls, women who attempted suicide were less likely to be married, employed, with post-secondary or higher education, and to have given birth in the previous two years, but more likely to have had a recent psychiatric hospitalization. Additionally, they were less likely to be current users of HC (15.6% *vs.* 22.2%), in particular of combined hormonal contraceptives (9.8% *vs.* 17.8%: ethinylestradiol (EE)-containing preparations, 7.5% *vs.* 13.9%; estradiol-containing preparations, 2.3% *vs.* 4.0%; *p* < 0.001) (Table 1). Specifically, current use of desogestrel and EE (0.9% *vs.* 2.0%, *p* = 0.045) and of drospirenone and EE (3.7% *vs.* 6.3%, *p* = 0.005) was less common among women who attempted suicide than in their controls (Table S3).

**Table 1:** Basic characteristics and HC use of the nested case–control study of attempted suicides

	Cases (N=818)		Controls (N=3272)		*p* value
	N	%	N	%	
**Marital status**					<0.001
Unmarried	667	81.5	2643	80.8	
Married	84	10.3	545	16.7	
Divorced	65	7.9	79	2.4	
Widowed	<5	NA	<5	NA	
Other	<5	NA	<5	NA	
**Socioeconomic group**					<0.001
Self-employed	19	2.3	96	3.0	
Upper-level employees	22	2.7	308	9.5	
Lower-level employees	118	14.5	850	26.2	
Manual workers	112	13.8	482	14.9	
Students	281	34.6	1067	32.9	
Pensioners	70	8.6	41	1.3	
Others	122	15.0	216	6.7	
Unknown	69	8.5	185	5.7	
**Education**					<0.001
Upper secondary	325	39.7	1488	45.5	
Post-secondary non-tertiary	<5	NA	15	0.5	
Short-cycle tertiary	6	0.7	39	1.2	
Bachelor	29	3.5	471	14.4	
Master	17	2.1	190	5.8	
Doctoral	<5	NA	12	0.4	
Missing (including, e.g., missing information on education other than of primary school level, school dropouts)	439	53.7	1057	32.3	
**Age group**^**a**^					1.000
15-19 years	249	30.4	996	30.4	
20-24 years	239	29.2	956	29.2	
25-29 years	143	17.5	572	17.5	
30-34 years	79	9.7	316	9.7	
35-39 years	56	6.8	224	6.8	
40-44 years	35	4.3	140	4.3	
45-49 years	17	2.1	68	2.1	
**Severe psychiatric disorder at baseline**	104	12.7	24	0.7	<0.001
**Chronic somatic disease at baseline**^**b**^	60	7.3	146	4.5	0.001
**Use of psychotropic medication**					
Antipsychotics	281	34.4	59	1.8	<0.001
Anxiolytics	186	22.7	31	0.9	<0.001
Hypnotics and sedatives	112	13.7	41	1.3	<0.001
Antidepressants	399	48.8	240	7.3	<0.001
Psychostimulants	13	1.6	13	0.4	<0.001
Psycholeptics and psychoanaleptics in combination	5	0.6	<5	NA	0.001
**Recent delivery**					<0.001
No	799	97.7	3077	94.0	
In the previous 6 months	< 5	NA	29	0.9	
6–24 months before	17	2.1	166	5.1	
**Recent hospitalization due to psychiatric disorders**					<0.001
No	189	23.1	2978	91.0	
In the previous 6 months	524	64.1	175	5.3	
6–24 months before	105	12.8	119	3.6	
**HC use in 2017**	344	42.1	1668	51.0	<0.001
**Current HC group**					<0.001
No HC	690	84.4	2545	77.8	
- Former use	233	28.5	1004	30.7	
- Never use	457	55.9	1541	47.1	
Current HC	128	15.6	727	22.2	
- Combined HC	80	9.8	584	17.8	
EE-containing combined HC	61	7.5	454	13.9	
Estradiol-containing combined HC	19	2.3	130	4.0	
- Progestin-only	48	5.9	143	4.4	

*EE* ethinylestradiol, *NA* not available

^a^matched by age

^b^Diabetes, multiple sclerosis, epilepsy, connective tissue diseases, ulcerative cholitis or Chron’s disease

Consistently, in the univariable logistic regression model, use of HC (OR 0.64, 95% CI 0.52–0.79), and specifically of combined HC (either EE-containing or estradiol-containing preparations) was associated with lower risk of AS compared to the risk in HC non-users (OR 0.50, 95% CI 0.39–0.64). No significant associations emerged with current use of progestin-only contraceptives (Table 2). The lower risk associated with combined HC (specifically, EE-containing contraceptives) remained significant after controlling for covariates (Models 1–2); however, when further adjusting for recent psychiatric hospitalizations and current use of psychotropic medications, no significant associations were detected between combined HC use and AS (Model 3, see Table 2). The results did not change in age-stratified analyses (Table S4). To exclude a healthy user bias, we repeated the analyses after distinguishing between former and never users (from 2017 onwards) of HC. Current use of HC was associated with lower odds of AS compared to never user after controlling for sociodemographic characteristics, but the association was not significant after adjustment for psychiatric diagnoses and recent delivery. Former use of HC was not associated with the risk of AS compared to never use (Table S5).

**Table 2:** Nested case–control study of AS. Odds ratios and 95% confidence intervals based on conditional logistic regression models

		Univariable		Model 1		Model 2		Model 3	
		Odds Ratio	95% CI	Odds Ratio	95% CI	Odds Ratio	95% CI	Odds Ratio	95% CI
Care Register for Health Care and Register of Primary Health Care visits data									
HC groups	No HC	Reference		Reference		Reference		Reference	
	Current HC	0.64	0.52–0.79	0.74	0.59–0.93	0.71	0.56–0.90	0.79	0.56–1.11
	Combined HC	0.50	0.39–0.64	0.59	0.45–0.77	0.55	0.42–0.73	0.68	0.45–1.02
	- EE-containing combined HC	0.49	0.37–0.64	0.56	0.41–0.75	0.54	0.40–0.73	0.74	0.47–1.17
	- estradiol-containing combined HC	0.53	0.33–0.87	0.71	0.43–1.20	0.60	0.34–1.05	0.50	0.21–1.19
	Progestin-only	1.24	0.88–1.74	1.40	0.96–2.06	1.39	0.94–2.05	1.08	0.62–1.88
Only Care Register for Health Care data									
HC groups	No HC	Reference		Reference		Reference		Reference	
	Current HC	0.59	0.48–0.73	0.65	0.51–0.82	0.65	0.51–0.82	0.86	0.61–1.21
	Combined HC	0.46	0.35–0.60	0.49	0.37–0.65	0.49	0.37–0.65	0.72	0.48–1.08
	- EE-containing combined HC	0.46	0.34–0.62	0.49	0.36–0.68	0.50	0.36–0.69	0.78	0.50–1.24
	- estradiol-containing combined HC	0.46	0.28–0.75	0.50	0.30–0.85	0.46	0.27–0.81	0.56	0.26–1.24
	Progestin-only	1.08	0.77–1.54	1.29	0.88–1.90	1.36	0.91–2.02	1.35	0.74–2.46

Usage of drugs in respect to the event day: no = no use in the past 180 days; current = use in the past 180 days. Multivariate Model 1 is adjusted with the following covariates: marital status, socioeconomic status, education; Model 2 is Model 1 further adjusted for chronic diseases (reimbursement codes^a^) and recent delivery; Model 3 is Model 2 further adjusted for recent psychiatric hospitalization and current use of psychotropic medications

*EE* ethinylestradiol, *HC* hormonal contraception

^a^diabetes, multiple sclerosis, epilepsy, severe psychiatric disorders, connective tissue diseases, ulcerative colitis or Crohn’s disease

In analyses stratified by psychiatric history, current use of HC, and in detail of EE-containing preparations, was associated with lower risk of AS only in women without psychiatric disorders. Among women with psychiatric disorders HC use was not associated with AS risk. The results did not change when comparing current use *vs.* former use *vs.* never use (Table 3).

**Table 3:** Associations between current HC use and attempted suicide in analyses stratified by psychiatric history

		No psychiatric history		Psychiatric history	
		Odds ratio	95% CI	Odds ratio	95% CI
HC groups	No HC	Reference		Reference	
	Current HC	0.73	0.58–0.91	0.78	0.50–1.21
	Combined HC	0.57	0.44–0.75	0.64	0.37–1.11
	- EE-containing combined HC	0.54	0.40–0.73	0.63	0.33–1.19
	- Estradiol-containing combined HC	0.69	0.41–1.16	0.68	0.25–1.84
	Progestin-only	1.41	0.96–2.06	1.10	0.54–2.23
	Never use	Reference		Reference	
	Former use	0.92	0.75–1.12	0.84	0.58–1.24
	Current use	0.71	0.56–0.90	0.74	0.46–1.18

Model controlled for marital status, socioeconomic status, education, chronic somatic diseases^a^ and recent delivery

*EE* ethinylestradiol, *HC* hormonal contraception

^a^diabetes, multiple sclerosis, epilepsy, connective tissue diseases, ulcerative colitis or Crohn’s disease

**Fig. 1:**
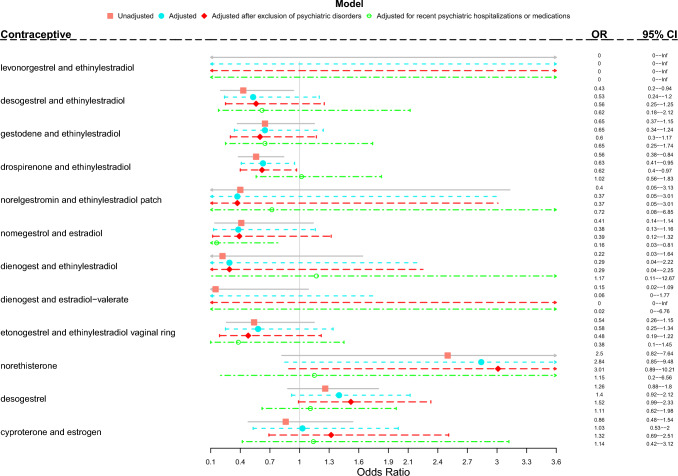
Associations between attempted suicide and current use (in the previous 180 days) of hormonal contraceptives. Results are expressed as Odds Ratios with 95% Confidence Intervals. For each substance the reference category is no use of the same substance in the 180 days before the attempted suicide. Adjusted model is controlled for marital status, socioeconomic status, education, chronic diseases and recent delivery. One substance in model a time. Care Register of Health Care and Register of Primary Health Care visits data together

In detail, in unadjusted models current use of combined oral contraceptives containing desogestrel and EE, and drospirenone and EE was associated with lower AS risk than non-use of the same preparations (OR 0.43, 95% CI 0.20–0.94; and OR 0.56, 95% CI 0.38–0.84, respectively). The association with drospirenone and EE remained significant after controlling for background characteristics (socioeconomic status, marital status, education, chronic diseases, recent delivery) and after exclusion of women with previous severe psychiatric disorders, but was not significant when further adjusting for recent psychiatric hospitalizations and use of psychotropic medications (Fig. 1).

In sensitivity analyses including only AS cases derived from specialist health care and recorded in the Care Register for Health Care, among the 1,174,475 person-years cumulated 760 AS cases were observed (IR 0.65 per 1000 person-years, 95% CI 0.60–0.70) (Table S6). Basic characteristics of the population of the nested-case control study based on Care Register for Health Care cases only are reported in Table S7 and Table S8. Associations with AS risk were similar to those in the original population (Table 2, Figure S1). In addition, current use of norethisterone-only oral contraceptives was associated with higher AS risk when controlling for background characteristics (OR 4.78, 95% CI 1.34–21.72) and after exclusion of women with previous severe psychiatric disorders (OR 5.63, 95% CI 1.46–21.73), but not when adjusting for recent psychiatric hospitalizations and use of psychotropic medications (OR 1.40, 95% CI 0.20–9.63) (Figure S2).

To specifically examine the risk of using HC in a high-risk group, the population of women with a history of previous suicide attempts (N = 889) was selected as an alternative sampling frame for the nested case–control study. In this population we observed 162 cases of attempted suicide during the follow-up period. For each case, four birth-year matched controls were selected (with the exception of two cases with only one available control), resulting in a final population of 808 women (646 controls, 162 cases). Results of unadjusted conditional regression models indicate that the use of any type of HC was not associated with the risk of (repeated) AS in this population (Table S9).

## Discussion

The main finding of our study is that current HC use was not significantly associated with an increased risk of attempted suicide in women of fertile age, even when considering both current and former use, and even in a high-risk population of previous suicide attempters. In addition, the current use of combined HC, in particular that of EE-containing preparations, was associated with a lower risk of attempted suicide compared to non-use of HC even after controlling for covariates in women without psychiatric disorders.

Our finding that the current use of HC is not significantly associated with an increased risk of AS in fertile-aged women contrasts with results reported by recent studies in the Nordic countries. A Danish study found a higher risk of the first AS in women aged 15–33 years using HC both currently (relative risk, RR 1.97, 95% CI 1.85–2.10) and previously (RR 3.40, 95% CI 3.11–3.71) compared with never-users [5]. Similarly, a large Swedish register-based study of women aged 15–22 years found an increased risk of suicidal behaviour (attempted or completed suicide) in users of combined oral contraceptives (HR 1.36, 95% CI 1.18–1.56) and progestin-only pills (HR 1.75, 95% CI 1.44–2.12) [6]. Further, two Swedish studies found a higher use of antidepressants among young (16–31 years, and especially in the youngest age-groups) users of progestin-only contraceptives, in particular of non-oral methods such as intrauterine systems, implants, injections, and transdermal patches, compared with users of combined hormonal contraceptives [22, 23]. A Korean study also reported positive associations between use of oral contraceptives and suicidality (ideation and attempt) in women over 20 years of age; however, the latter compared, with a retrospective design, lifetime prevalence of oral contraceptive use and period prevalence of suicidality, and did not provide any information on the type of oral contraceptives or on other types of HC [24]. One of the plausible reasons underlying these different findings is the inclusion of older women in our sample, where the prevalence of AS in general is low and who usually have a distinct profile of HC use. In this regard, it must be acknowledged that older women are more likely to use long-active reversible contraceptive methods, such as the levonorgestrel-releasing intrauterine system, which were not all included in our study, given that most of these methods are provided free-of-charge by some municipalities in Finland and can be used for up to five years. However, our results did not change in age-stratified analyses. Another possible explanation to our findings is that women with psychiatric disorders may be less likely to use HC [16], but more likely to attempt suicide. However, no associations between HC use and a higher risk of AS emerged in analyses stratified by psychiatric history, nor in the high-risk population of previous suicide attempters. While these results have clinical implications as they suggest that HC does not represent a risky prescription in this population, it remains however possible that inadequate power partly accounts for lack of a significant association, especially concerning progestin-only contraceptives, which are known to have the worst effects on mood and were in fact related to a higher proportion of suicide attempts in the current study.

The observation of a negative association between current use of EE-containing preparations and AS risk is a novel one, although driven substantially by the group of women without any psychiatric disorders. Overall, these results are in line with those of a recent Swedish register-based study conducted on a selected population of over 20,000 women with premenstrual disorder, which suggested that use of HC, and in particular of combined HC, was associated with no or a lower risk of AS in women with as well as without psychiatric disorders [14]. On the other hand, Skovlund et al. [5] found an increased risk associated with the use of all EE-containing combined oral contraceptives, but the highest risk was seen in users of patch, vaginal ring, and progestin-only products. Differently from the Danish study, we controlled our results for the current use of any psychotropic medications, including antipsychotics and anxiolytics in addition to antidepressants. This may be relevant, as we have previously found positive associations between HC use and the use of psychotropic drugs of any class [25]. The opposite findings may also reflect the effect of unknown confounding factors that cannot be easily uncovered in register-based studies. Similarly as Skovlund et al. [5] we also found a tendency for a higher AS risk with the use of progestin-only products, such as norethisterone and desogestrel containing oral contraceptives. However, in contrast with the Danish results, we did not find associations between the risk of AS and the use of vaginal ring or patch. Even though the negative impact of norethisterone on mood has been observed previously [26, 27], our results need caution, given that the use of norethisterone-only pills in Finland is rather limited (Table S1). In general, the use of progestin-only methods is not contraindicated during lactation; however, the tendency did not change after controlling for a recent delivery.

Another key finding of our study is a tendency for a lower AS risk in women using drospirenone and EE. Drospirenone has progestogenic, antimineralcorticoid and antiandrogenic activities. These characteristics are thought to contribute to its favourable influence on mood also in women suffering from premenstrual dysphoric disorder [28–31]. This finding is of relevance, as oral contraceptives containing drospirenone (and third-generation progestins such as desogestrel, etonogestrel, and gestodene) are used more commonly in Finland than in the other Nordic countries, and oral contraceptive containing drospirenone is the most used combined oral contraceptive in Finland (Table S1).

Our study has some limitations. By using registry data derived mainly from specialist healthcare, we were not able to detect less severe cases of AS that remained unknown to the healthcare system, which likely account for almost half of the self-reported AS [32]. Similarly, we were not able to distinguish between attempted suicide and other types of suicidal and self-harm behaviours (superficial self-harm, stress-relieving self-harm, cry for help, etc.), since we did not have access to individual medical records. Additionally, as HC use was defined as redeemed prescriptions rather than on its actual use monitored in clinical practice, misclassification cannot be ruled out. However, because the Social Insurance Institution of Finland does not reimburse HC, it is likely that most who purchased the drug did in fact use it. On the one hand, by using at least two redeemed HC prescriptions during the 180-day period as the criterion for current HC use, we cannot exclude a null finding driven by the inclusion of HC users in the non-user group. On the other hand, it cannot be excluded that women currently not using HC at the time of the AS (during the past 6 months) were in fact former users, who discontinued their contraceptive due to side effects. For example, Skovlund et al. [5] found a higher risk of AS in former users than in current users of HC. However, a recent study found only marginal increase in the risk estimates for depression when using never-users compared to non-users of HC as the reference category [33]. Importantly, our results did not change when splitting the HC non-user category into former and never-users. Because records of HC use are included in the Finnish register only starting from 2017, a longer follow-up period was not possible. Another limitation arises from the lack of information on the precise contents of the contraceptive preparations used, which precluded any analyses on the effect of different doses of EE. Additionally, we lacked information on the use of non-hormonal methods (e.g., copper intrauterine device, and barrier methods) as well as contraceptives obtained free-of-charge as part of municipal programs, concerning especially long-active reversible contraceptive methods (in particular the hormonal intrauterine system). Further, we managed to capture a nearly complete proportion of those women having a history of psychiatric disorders with a diagnosis from specialised healthcare, reimbursement rights and redeemed psychotropic medications, although some patients may have contacted only primary healthcare and not claimed their special reimbursement rights. Finally, we cannot exclude that the detected associations are confounded by external unaccounted factors.

Among the strengths of our study is the use of Finnish register data of proven high quality [34], and the identification of AS cases based on the diagnostic ICD-10 codes from specialised healthcare from 2018 to 2019. The nested case–control design we used produces unbiased estimates and is free from weaknesses of the ordinary case–control design. It uses correct sampling of controls that accounts the follow-up time [35, 36], and appears more straightforward than a cohort design with respect to recent, non-cumulative exposures. In addition, the control women were matched by age.

Taken together, our results convey the reassuring message to fertile-aged women seeking contraception that current HC use was not significantly associated with an increased risk of attempted suicide. At the same time, they once more stress the importance of a personalized choice of the best and safest contraceptive option, which should include the assessment of mental health status and suicide risk.

## Supplementary Information

Below is the link to the electronic supplementary material.

Supplementary file1 (PDF 7 kb)

Supplementary file2 (PDF 5 kb)

Supplementary file3 (DOCX 55 kb)

## Data Availability

The data that support the findings of this study are available from Statistics Finland, the Finnish Institute for Health and Welfare, and the Social Insurance Institution, but restrictions apply to the availability of these data, which were used under license for the current study, and so are not publicly available. Data are, however, available from the authors upon reasonable request and with permission of FinData (https://www.findata.fi/en/).
