# Supplementary figures and images for: Use of systemic hormonal contraception and risk of attempted suicide: a nested case–control study
Source: Eur J Epidemiol. 2024 Sep 3;39(9):1013–22. doi: 10.1007/s10654-024-01155-z (PMC11470879; doi:10.1007/s10654-024-01155-z)

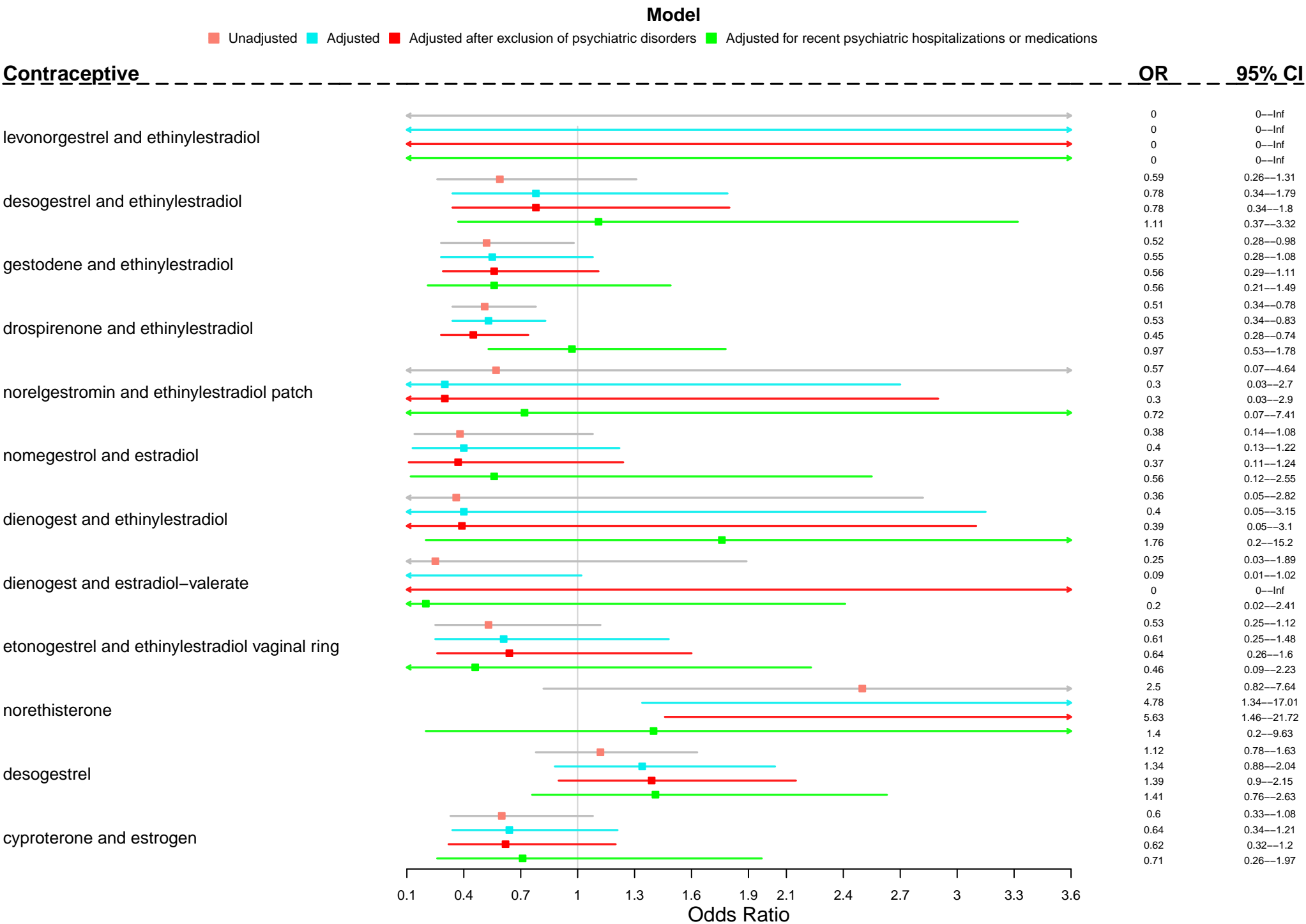

Supplement: Supplementary file 1 — Supplementary file1 (PDF 7 kb) [file 10654_2024_1155_MOESM1_ESM.pdf]

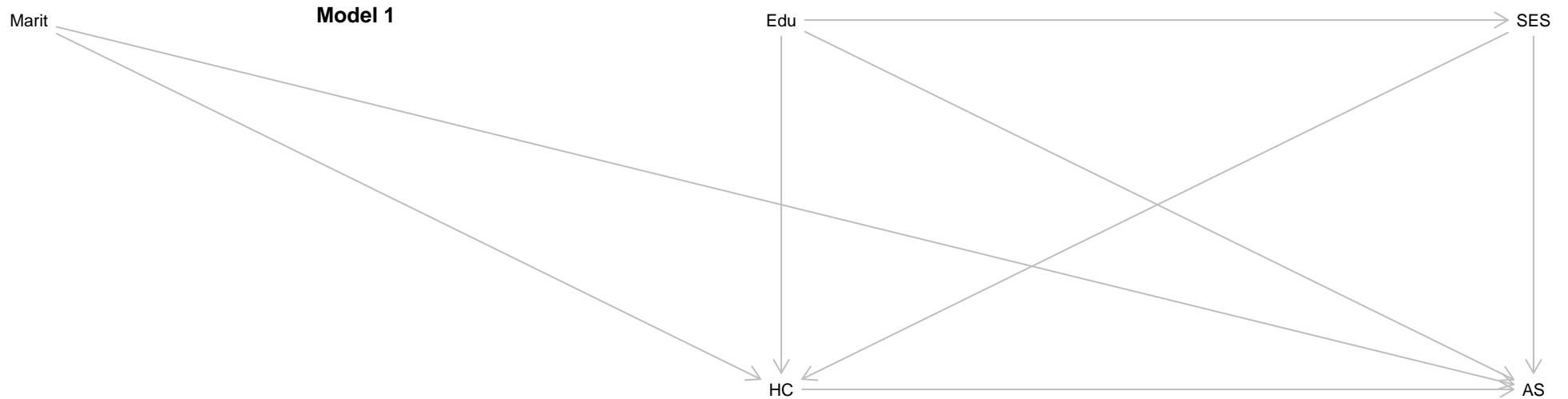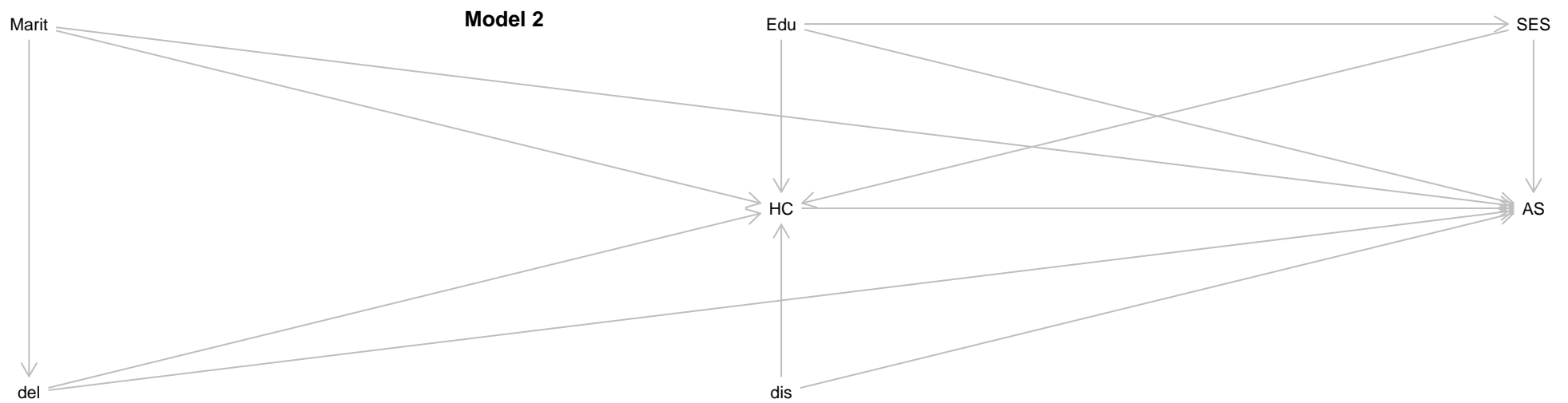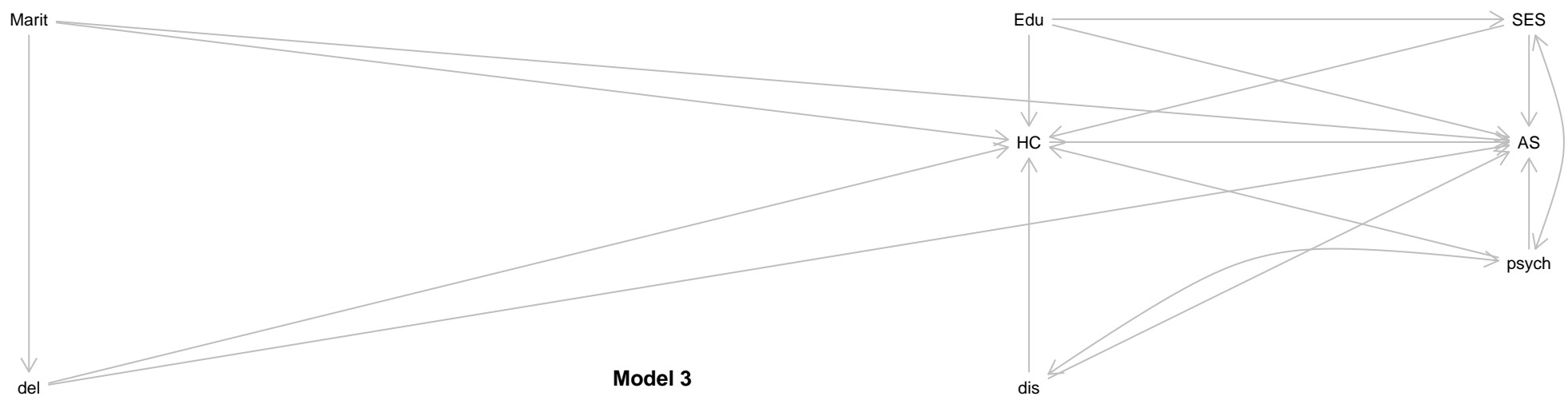

Supplement: Supplementary file 2 — Supplementary file2 (PDF 5 kb) [file 10654_2024_1155_MOESM2_ESM.pdf]
